# Methods of terms formation in nuclear medicine

**DOI:** 10.12688/openreseurope.18941.1

**Published:** 2025-02-10

**Authors:** Nigar Sadigova, Salmi Aliyeva

**Affiliations:** 1 Azerbaijan University of Architecture and Construction, Baku, Azerbaijan; 2 Institute of Linguistics named after Nasimi, Baku, Azerbaijan; 3 DeepTechLab, Lisbon, Portugal

**Keywords:** Nuclear medicine, Prefixes and Suffixes, Abbreviations and Acronyms, Descriptive Compounding, Terminology

## Abstract

**Background:**

Nuclear medicine is a dynamic field that uses radioactive substances for diagnosis, therapy, and research. Developing terminology in this domain involves addressing complex concepts across multiple disciplines. Greek and Latin roots provide universal terms, enabling clear communication among global professionals. Scientific prefixes, suffixes, and abbreviations further simplify advanced imaging concepts, while descriptive compounding and eponyms create intuitive and historically relevant terms.

**Methods:**

The creation of nuclear medicine terminology combines linguistic precision with scientific innovation. Greek and Latin roots form universally understood terms with historical significance. Prefixes and suffixes add precision to describe technologies, while abbreviations like PET (Positron Emission Tomography) simplify complex terms for efficient communication. Descriptive compounding explains mechanisms clearly, and naming conventions honor inventors, adding historical depth. Standard units like Becquerel (Bq) and descriptors like SUV ensure consistent measurements. Terminology adapts to advances like PET/MRI, merging functional and anatomical imaging for enhanced clarity and utility.

**Results:**

The systematic approach to nuclear medicine terminology provides a clear and consistent framework for communication. Greek and Latin roots standardize terms for complex processes, while prefixes and suffixes ensure descriptive precision. Abbreviations like PET and SUV improve communication speed and efficiency. Descriptive compounding simplifies understanding of technologies, and historical naming conventions honor significant contributions. Standard units, such as Bq and Gy, ensure measurement consistency. Hybrid imaging systems like PET/MRI drive terminology evolution, reflecting technological integration and enhancing diagnostic and therapeutic precision.

**Conclusion:**

Nuclear medicine’s systematic terminology ensures clarity and precision, vital for advancing diagnostics, treatment, and research. By combining classical roots, scientific prefixes, and standardized units, this framework fosters global collaboration and innovation. Abbreviations and intuitive compounding enhance accessibility, while eponyms honor historical contributions. As hybrid imaging systems evolve, this adaptable terminology continues to support innovation, bridging complex concepts with practical applications to advance patient care.

## Plain language summary

This article explains how terms in nuclear medicine are created and why they’re essential for clear communication in this field. Nuclear medicine is a specialized area that uses radioactive substances for diagnosing and treating diseases. Because this field deals with complex technology and science, it needs a precise language that professionals worldwide can understand. To achieve this, the terms often use roots from Greek and Latin, scientific prefixes and suffixes, and abbreviations. For example, "tomography" combines Greek words meaning "slice" and "writing," describing how imaging shows “slices” of the body.

Additionally, acronyms like PET (Positron Emission Tomography) make it easier to discuss complex processes quickly. The article also highlights how some terms honor inventors, like "Curie," a unit of radioactivity named after Marie Curie. Standard scientific units and descriptors (such as the Standardized Uptake Value) provide a universal way to measure and interpret results, enhancing collaboration and patient care. These well-thought-out naming methods make nuclear medicine more accessible, consistent, and effective in advancing global health.

## Introduction

Nuclear medicine is a dynamic and rapidly advancing discipline that employs radioactive materials for diagnostic, therapeutic, and research applications. As new technologies and imaging techniques emerge ^1^, the demand for precise and universally comprehensible terminology becomes increasingly vital. The creation of terms in nuclear medicine is not just a matter of language; it represents the complexity of the field and the need for effective communication among professionals from various specialties. The methodology used to develop these terms encompasses several fundamental approaches. Many medical terms are grounded in classical language roots, particularly Greek and Latin, providing a coherent and etymological framework that conveys complex concepts in a way that is widely recognized. This classical basis enables practitioners to express intricate ideas related to imaging techniques and subatomic particles effectively, fostering collaboration across disciplines and enhancing overall comprehension.

Moreover, the integration of scientific prefixes and suffixes is crucial for formulating precise and descriptive terminology. By using familiar linguistic elements, professionals can clarify the nature and function of medical technologies and processes, ensuring consistency across diverse fields. This systematic approach not only facilitates effective communication but also promotes clarity and accuracy, which are essential in a discipline where precision is critical. Abbreviations and acronyms have become vital tools in nuclear medicine, striking a balance between brevity and meaning in a fast-paced setting. Terms such as PET (Positron Emission Tomography) and SUV (Standardized Uptake Value) illustrate how complex terminology can be simplified into easily recognizable forms, allowing for efficient communication in clinical and research environments. These linguistic strategies enhance clarity and promote collaboration among professionals from varied backgrounds, ultimately leading to improved patient care.

Additionally, the practice of descriptive compounding enables the formation of intuitive terms that effectively communicate the purposes and mechanisms of complex technologies. By combining simple, familiar words, professionals can quickly understand intricate concepts, thereby enhancing interdisciplinary cooperation and furthering the field. As nuclear medicine continues to develop, the incorporation of technological advancements and the evolution of terminology will be essential. This introduction lays the groundwork for a comprehensive examination of the methodologies involved in forming terms in nuclear medicine, emphasizing their significance in promoting clear, accurate, and effective communication within this ever-evolving field.

## Methods

Nuclear medicine terminology heavily relies on Greek and Latin roots, which provide a universal and systematic linguistic framework. These classical roots ensure consistency and clarity, enabling terms to be easily translated and understood across cultures and disciplines. For example, tomography derives from the Greek words tomos (slice) and graphia (writing), aptly describing imaging techniques that visualize cross-sections of the body. Similarly, positron, derived from the Latin positus (positive), reflects the particle’s charge and role in PET imaging, enhancing communication about the underlying physics of these technologies. The use of these roots ensures that terms are both descriptive and historically grounded, supporting global collaboration in nuclear medicine.

The combination of scientifically meaningful prefixes and suffixes forms precise and descriptive terms in nuclear medicine. For instance, radiopharmaceutical combines the Latin prefix radio- (radiation) and the Greek suffix -pharmaceutical (medicine) to describe radioactive drugs used for imaging or therapy. Similarly, isotope, derived from the Greek iso- (equal) and -topos (place), explains elements with the same atomic number but different neutron counts. This method allows for intuitive terminology that categorizes functions and properties of substances and technologies, facilitating interdisciplinary communication and the creation of new terms as the field advances.

Abbreviations and acronyms simplify complex terms without losing precision, aiding efficiency in communication. Widely used acronyms such as PET (Positron Emission Tomography) and SUV (Standardized Uptake Value) encapsulate detailed processes while enabling quick and clear understanding. Acronyms like FDG (Fluorodeoxyglucose) and CT (Computed Tomography) standardize terminology globally, supporting collaboration in education, clinical practice, and research. This practice reduces ambiguity, enhances accessibility, and ensures consistent usage across disciplines and regions.

Descriptive compounding combines familiar words to create terms that intuitively describe functions, such as coincidence detection in PET or half-life for radioactive decay. Additionally, naming terms after contributors, such as the Anger camera or Curie, honors their legacy while embedding historical significance in scientific language. These methods provide clarity, accessibility, and adaptability, ensuring the terminology evolves alongside technological advancements while maintaining a connection to the field's roots in innovation and discovery.

### Classical language roots (Greek and Latin)

The formation of scientific and medical terms has historically relied on Greek and Latin, two classical languages that form the bedrock of terminological construction across various disciplines. These roots offer a universal, systematic, and precise way to describe complex medical processes and technologies, especially in specialized fields like nuclear medicine. The reliance on Greek and Latin allows for consistency and clarity in terminology, ensuring that terms are comprehensible and easily translatable across linguistic and cultural boundaries. Moreover, these classical roots provide a logical structure to terms, where the origin of each word element carries significant meaning that contributes to a better understanding of the technology or process it describes.

In nuclear medicine, Greek and Latin roots are frequently employed to articulate the principles of imaging and diagnostic techniques in a way that reflects their technical function. For instance, the term **tomography** is derived from the Greek words “tomos” (meaning "slice" or "cut") and “graphia” (meaning "writing" or "description"). The concept of "writing by slices" or imaging by sections is fundamental to medical imaging techniques ^2^, including PET, CT, and MRI scans, all of which involve creating detailed cross-sectional images of the body. This terminology elegantly captures the essence of the scanning process, where the body is visualized in thin slices to detect anomalies, diseases, or abnormalities that may not be visible in standard two-dimensional imaging techniques.

Another example is **positron**, derived from the Latin root “positus,” meaning "placed" or "positive," which directly refers to the subatomic particle that plays a critical role in PET (Positron Emission Tomography) imaging. A positron is the antimatter counterpart of the electron, having the same mass but a positive charge. When a positron is emitted by a radioactive tracer within the body, it interacts with an electron, resulting in the annihilation of both particles and the release of gamma photons. These photons are subsequently detected by the PET scanner, allowing for the creation of an image based on the metabolic activity within the body. By incorporating the Latin term for "positive," the word positron directly reflects the particle’s charge and its role in the PET scanning process, facilitating clear communication about the underlying physics of the imaging technology ^3^.

The use of classical roots in nuclear medicine extends beyond these two examples, serving as a unifying framework for naming techniques, substances, and phenomena. This linguistic practice is not only a means of standardization but also contributes to the rich historical continuity of scientific language, linking modern medical innovations to the centuries-old traditions of inquiry and knowledge-sharing in the fields of physics, chemistry, and biology. Greek and Latin provide a shared linguistic foundation that transcends the technical complexities of the field, making it accessible to an international community of practitioners and researchers while ensuring that the terms are descriptive and functionally accurate.

### Combination of scientific prefixes and suffixes

The formation of medical and technical terms often relies on the combination of scientifically meaningful prefixes and suffixes to convey the nature, function, or purpose of the technology or process being described. This methodological approach allows for precision, clarity, and consistency across various disciplines, particularly in the complex and specialized field of nuclear medicine. By using well-established linguistic building blocks, new terms can be systematically crafted to provide immediate insights into the underlying concepts or mechanisms they represent. Such combinations help standardize terminology, making it accessible and understandable to professionals across different fields, from medicine to physics, and ensuring seamless communication.

In nuclear medicine, prefixes and suffixes frequently serve to categorize and clarify the function of various substances or techniques. For instance, the term **radiopharmaceutical** is a compound word that combines the prefix "radio-" and the suffix "-pharmaceutical." The prefix "radio-" is derived from the Latin word "radius," meaning "ray" or "radiation," and in this context, it refers to radioactive substances. The suffix "-pharmaceutical," which comes from the Greek word "pharmakeutikos" (pertaining to drugs), describes a substance used for medical purposes. When combined, the term radiopharmaceutical refers to a class of drugs that contain radioactive isotopes, specifically designed for diagnostic imaging or therapeutic applications. In Positron Emission Tomography (PET) and other forms of nuclear medicine, radiopharmaceuticals such as fluorodeoxyglucose (FDG) play a crucial role in highlighting metabolic processes in the body by emitting radiation that can be captured by imaging devices. The construction of the term directly reflects its dual nature as both a radiation-emitting agent and a pharmaceutical product, making it immediately comprehensible to those familiar with the scientific language.

Another illustrative example is the term **isotope**, which is derived from the Greek prefix "iso-" meaning "equal" and "topos" meaning "place." This term was coined to describe elements that occupy the same position on the periodic table (i.e., have the same atomic number) but differ in their neutron count, leading to variations in atomic mass. In nuclear medicine, isotopes are integral to the functioning of imaging modalities like PET, where radioactive isotopes such as Fluorine-18 (F-18) are used as tracers. These isotopes decay by emitting positrons, which are detected and visualized by the PET scanner. The term isotope captures the fundamental nature of these elements—having an identical position on the periodic table but differing in other properties—thus providing a clear and concise label for this essential concept in nuclear medicine and related fields.

The combination of scientific prefixes and suffixes not only defines the nature of medical substances and technologies but also categorizes them according to their specific function or effect. This method ensures that terms are universally applicable, reducing ambiguity and enabling more accurate communication across various branches of science and medicine. For instance, terms like **radiobiology** (the study of the biological effects of radiation) or **thermonuclear** (relating to nuclear reactions at high temperatures) follow similar construction patterns, combining meaningful prefixes and suffixes that allow for a clear understanding of the subject matter. This uniform approach to terminology formation is especially important in interdisciplinary fields like nuclear medicine, where concepts from physics, chemistry, and biology intersect.

In addition to clarity, this systematic combination of linguistic elements enables the creation of new terms as scientific knowledge evolves. As new technologies or processes are developed, new terms can be created by adapting existing prefixes and suffixes, ensuring that the language of the field keeps pace with advancements. This approach not only reflects the functional properties of the new technology but also maintains continuity with existing terminology, reinforcing the logical structure of the field’s language.

### Abbreviations and acronyms

In technical fields such as nuclear medicine, where precision and efficiency in communication are critical, the use of abbreviations and acronyms is a standard practice. These shortened forms of longer, descriptive terms allow for the simplification of complex language without sacrificing the depth or accuracy of the concepts they represent. By condensing multi-word phrases into easily recognizable acronyms, professionals in the field can streamline verbal and written exchanges, improving the speed and clarity of communication while maintaining the full meaning of the original terms. This practice is particularly valuable in multidisciplinary settings, where medical professionals, physicists, engineers, and researchers must collaborate effectively, often across linguistic and cultural boundaries.

In nuclear medicine, **PET** is one of the most commonly used acronyms. It stands for **Positron Emission Tomography**, an advanced imaging modality that provides insight into metabolic processes within the body by detecting the emission of positrons from radioactive tracers. While the full term precisely describes the technique's underlying mechanism—positron emission and tomography (imaging by slices)—the acronym PET allows for rapid communication among professionals. Despite its brevity, the acronym carries with it a well-defined set of associations and implications regarding the technology's purpose, function, and application in both diagnostic and research contexts. PET is used extensively to detect cancers, assess heart conditions, and investigate neurological disorders, and its acronym has become so well-recognized that it requires no further explanation among medical and scientific communities. This abbreviation thus serves not only as a convenient shorthand but also as a symbol for a widely utilized and understood diagnostic tool.

Similarly, the acronym **SUV** stands for **Standardized Uptake Value**, a key parameter in PET imaging that quantifies the uptake of radiopharmaceuticals by tissues. The SUV is crucial for evaluating metabolic activity, as it allows practitioners to compare the concentration of the tracer in a specific region of interest against the expected norm for that tissue type. This measurement plays an essential role in identifying abnormal tissue activity, such as that seen in tumors, and in monitoring the effects of treatments over time. While the concept of standardized uptake value is complex, involving mathematical calculations and normalization techniques, the acronym SUV simplifies communication about this critical metric. By adopting this abbreviation, professionals can focus on interpreting the value itself rather than repeatedly explaining the underlying concept, thus facilitating more efficient discussions in both clinical and research settings.

Beyond PET and SUV, the use of abbreviations and acronyms is widespread in nuclear medicine due to the inherently interdisciplinary nature of the field. Terms that originate in physics, chemistry, or biology often have to be adapted for use in a medical context, resulting in lengthy compound phrases that describe the scientific processes in detail. Acronyms provide a solution to this issue by compressing the complexity into manageable units. For example, **FDG**, which stands for **Fluorodeoxyglucose**, is the most commonly used radiotracer in PET imaging. While "fluorodeoxyglucose" accurately reflects the chemical structure and function of the tracer—a glucose analog tagged with the radioactive isotope fluorine-18—the acronym FDG simplifies its usage in medical reports, discussions, and research papers. Similarly, **CT** (Computed Tomography), **MRI** (Magnetic Resonance Imaging), and **SPECT** (Single-Photon Emission Computed Tomography) are widely used acronyms that represent other key imaging modalities, each with a specific role in diagnostics. These acronyms not only facilitate interdisciplinary communication but also enhance the accessibility of advanced imaging techniques to non-specialists.

The use of abbreviations and acronyms also plays a significant role in enhancing efficiency in education, research, and clinical practice. Medical students, residents, and even experienced professionals must navigate a vast array of terms and concepts in nuclear medicine, and acronyms help them retain and recall key information more easily. For instance, instead of memorizing the full names and mechanisms of all the radiopharmaceuticals used in PET, students can focus on understanding the different properties of **FDG**, **NaF** (Sodium Fluoride), or **Ga-68** (Gallium-68), abbreviations that encapsulate the essence of these substances. Similarly, in research papers and clinical reports, where space and time are often at a premium, acronyms allow for concise yet informative communication, ensuring that the content is both dense in information and easy to digest.

Furthermore, the standardization of abbreviations and acronyms across the medical and scientific fields promotes consistency and reduces the likelihood of errors. Acronyms such as PET, SUV, FDG, and CT are universally recognized, ensuring that professionals in different parts of the world, or even those from different medical disciplines, can understand and interpret these terms in the same way. This global standardization is especially important in an era where medical knowledge is shared across borders through research publications, international conferences, and global health initiatives. The use of universally accepted abbreviations ensures that findings and innovations in nuclear medicine can be communicated and implemented across different healthcare systems without the risk of misinterpretation.

### Descriptive compounding

In the realm of nuclear medicine, descriptive compounding serves as a linguistic technique for constructing terms that clearly and intuitively convey the function or purpose of complex technologies and processes. This approach involves combining simpler, easily recognizable words or word elements into compound terms that accurately describe the technology or its mechanism. By doing so, descriptive compounding helps to demystify sophisticated concepts, making them more accessible to both specialists and non-specialists alike. In technical fields where precision and clarity are paramount, such terms play a crucial role in enabling efficient communication and a deeper understanding of the tools and techniques in use.

One of the clearest examples of descriptive compounding in nuclear medicine is the term **coincidence detection**, a process essential to Positron Emission Tomography (PET). This term is a compound of two words: "coincidence," meaning the occurrence of two events at the same time, and "detection," which refers to the act of identifying or discovering something. In the context of PET imaging, coincidence detection describes the technology’s ability to detect two gamma photons that are emitted simultaneously from a positron-electron annihilation event. When a positron, emitted by a radioactive tracer, interacts with an electron, both particles are annihilated, producing two gamma photons that travel in opposite directions. By detecting these two photons at the exact same moment (in coincidence), the PET scanner can accurately determine their point of origin inside the body, allowing for precise imaging of metabolic activity. The term "coincidence detection" effectively communicates this process by directly referencing the two key aspects: the simultaneity of photon emissions and the detection mechanism used by the scanner. This compound term not only captures the essence of the process but also simplifies it for those unfamiliar with the underlying physics.

Another prime example of descriptive compounding is **time-of-flight (TOF)** ^4^, a technique used to improve the spatial resolution and accuracy of PET imaging. The term combines "time," referring to the measurement of a temporal interval, and "flight," which suggests motion through space. In nuclear medicine, TOF refers to the measurement of the time difference between the detection of two gamma photons produced in a positron-electron annihilation event. By calculating the slight difference in the arrival times of the photons at the PET scanner’s detectors, the system can pinpoint the exact location of the annihilation event more accurately. This added precision enhances the image quality and diagnostic capabilities of PET scans. The term "time-of-flight" intuitively describes the process: the measurement of time in relation to the movement (or flight) of photons. Through descriptive compounding, TOF succinctly conveys a sophisticated concept without relying on overly technical jargon, making it easier to grasp for those less familiar with the physics behind the technology.

Descriptive compounding is not limited to PET imaging but extends across a wide range of nuclear medicine technologies. For instance, the term **radiotracer** is another example of compounding, combining "radio," referring to radioactive materials, and "tracer," indicating a substance that follows or traces a specific biological pathway. In nuclear medicine, radiotracers are substances that emit radiation as they move through the body, allowing for the imaging of physiological processes. The term radiotracer effectively conveys its function—the tracking of radioactive substances—offering a clear understanding of its use in diagnostic imaging, particularly in PET and Single-Photon Emission Computed Tomography (SPECT).

Additionally, the term **half-life** is a well-known compound term frequently used in both nuclear medicine and physics. It combines "half," meaning one of two equal parts, and "life," in this case referring to the duration over which a radioactive substance remains active. The term describes the amount of time it takes for half of the atoms in a radioactive sample to decay. In nuclear medicine, understanding the half-life of a radiotracer is critical for determining how long the substance remains effective for imaging and when it will be safely eliminated from the body. The compound term half-life intuitively captures the temporal aspect of radioactive decay, offering a simple yet powerful descriptor for a complex concept.

Descriptive compounding, therefore, not only serves the practical purpose of simplifying terminology but also enhances the intuitiveness of scientific language. By fusing familiar words to form new terms that accurately reflect the function or purpose of technologies, this method makes complex concepts more understandable. Furthermore, descriptive compounds ensure that the terms remain transparent, allowing those who may not have an extensive background in nuclear physics or radiochemistry to comprehend the mechanisms at play. This accessibility is particularly important in interdisciplinary fields like nuclear medicine, where professionals from diverse backgrounds, including doctors, physicists, engineers, and technologists, must communicate and collaborate effectively.

Moreover, the process of descriptive compounding provides flexibility and adaptability to scientific language. As new technologies and processes are developed, new compound terms can be created using this method, ensuring that the language evolves alongside technological advancements. For example, with the ongoing development of hybrid imaging systems, terms such as **PET/CT** and **SPECT/CT** have emerged, combining modalities from two different imaging techniques (PET and CT or SPECT and CT). These compound terms immediately inform the user that the technology involves the integration of two distinct imaging methods, enhancing diagnostic precision and allowing for more comprehensive assessments of both anatomical structure and physiological function.

### Named after discoverers or developers

In scientific fields, including nuclear medicine, it is common for certain technologies, processes, or discoveries to be named after the individuals who pioneered or significantly contributed to their development. This naming convention serves as both an acknowledgment of the individual's contribution and a means of memorializing their work within the scientific community. By attaching a discoverer’s or developer’s name to a particular technique or device, the term not only reflects historical context but also emphasizes the importance of personal innovation in driving technological advancements. In nuclear medicine, this practice highlights the role that individual scientists and inventors have played in shaping the field, contributing breakthroughs that have had lasting impacts on diagnostics and treatment.

A prime example of this naming convention in nuclear medicine is the **cyclotron**, a type of particle accelerator named after its inventor, Ernest O. Lawrence. The cyclotron, first developed in the early 1930s, revolutionized nuclear physics and later became essential in the production of radioisotopes for medical imaging and cancer therapy. Lawrence's invention allowed for the acceleration of charged particles to high energies using a magnetic field, enabling scientists to produce a variety of isotopes that could be used in diagnostic imaging techniques, such as Positron Emission Tomography (PET). The naming of the cyclotron after Lawrence honors his contribution to the development of nuclear technology and underscores the centrality of this invention to the field of nuclear medicine. Cyclotrons are still widely used today to generate short-lived isotopes such as Fluorine-18, which plays a key role in PET imaging, allowing for detailed visualization of metabolic processes within the body.

Another notable example is the **Curie** (Ci) ^5^, a unit of radioactivity named after Marie and Pierre Curie, pioneers in the study of radioactivity. The unit is used to measure the activity of a radioactive substance, specifically the number of decays that occur per second. Marie Curie’s groundbreaking work in discovering radium and polonium, as well as her exploration of the therapeutic applications of radioactivity, laid the foundation for many of the principles that underpin modern nuclear medicine. The use of her name to denote a unit of radioactivity serves as a lasting tribute to her contributions to science and the medical field. It also reflects the deep connection between early research into radioactivity and the development of modern diagnostic and therapeutic techniques. Today, the Curie has been largely replaced by the Becquerel (Bq) in the International System of Units, but its legacy endures, particularly in medical contexts.

Additionally, the **Anger camera**, named after Hal O. Anger, is another example of a nuclear medicine technology named in honor of its inventor. The Anger camera, also known as the gamma camera, is a crucial device used in single-photon emission computed tomography (SPECT) and other nuclear imaging techniques. Developed in the 1950s, the gamma camera detects gamma radiation emitted from radiopharmaceuticals injected into the body, converting the radiation into an image that provides valuable information about the functioning of internal organs. Hal Anger’s innovation in designing this device had a profound impact on the field of nuclear medicine, as it allowed for non-invasive imaging of physiological processes, leading to better diagnostic capabilities. The Anger camera remains widely used today, and its name stands as a testament to the inventor's role in advancing medical imaging technology.

This practice of naming medical technologies after their developers not only honors the contributions of individual scientists but also provides a historical and personal dimension to the terminology used in the field. It creates a direct connection between the advancement of knowledge and the individuals responsible for those breakthroughs, allowing future generations of practitioners to appreciate the origins of the tools and techniques they use. This tradition also reflects the collaborative and cumulative nature of scientific progress, where each new invention builds on previous discoveries and innovations.

In some cases, the names of developers or discoverers become so deeply embedded in the terminology of nuclear medicine that their origins may be overlooked or forgotten by those who use them. For instance, **Geiger-Müller counters** ^6^, used to detect ionizing radiation, are named after Hans Geiger and Walther Müller, who collaborated in the development of this radiation detection device. Geiger’s earlier work on detecting alpha particles and his partnership with Müller in refining the technology led to the creation of a device that could detect not just alpha particles but also beta particles and gamma rays. The Geiger-Müller counter became a vital tool in nuclear research and medicine, allowing for the precise measurement of radiation exposure, which is critical in both diagnostic imaging and radiation therapy. While the term "Geiger counter" is widely recognized, its origins serve as a reminder of the contributions of individual scientists to the field of radiation detection and safety.

Named terms also play an important role in fostering a sense of continuity and tradition within the scientific community. They provide a way to celebrate the legacy of innovators whose work has shaped the current landscape of nuclear medicine. For students and professionals alike, learning about the origins of these terms can deepen their understanding of the historical context in which major discoveries were made and the challenges that early pioneers faced in advancing the field.

### Standard scientific units and terminology

In nuclear medicine, many terms are derived from universally accepted scientific principles and measurement systems, which provide a foundation of precision and consistency crucial for communication and practice. These terms often describe quantifiable properties and processes, making it possible to standardize information across different disciplines and geographic regions. By adhering to established scientific conventions, nuclear medicine ensures that professionals, regardless of their location or specialization, can interpret and apply key concepts accurately. This reliance on standard units and terminology is particularly important in a field that involves the precise manipulation of radioactive materials, advanced imaging techniques, and complex biological interactions. Among these standardized terms, **half-life** stands out as a foundational concept, deeply rooted in physics but essential to the safe and effective use of radiopharmaceuticals in medical applications.

**Half-life** refers to the time it takes for half of the atoms in a radioactive sample to decay, and it is critical for understanding the behavior of radioactive isotopes used in nuclear medicine. This term, derived from the principles of nuclear physics, provides a quantifiable measure of the rate at which a radiopharmaceutical loses its radioactivity over time. In the context of Positron Emission Tomography (PET), where radiopharmaceuticals are introduced into the body to trace metabolic processes, knowing the half-life of the substance is essential for several reasons. First, it determines the window of time in which the radiotracer remains active and can produce meaningful diagnostic images. Radiopharmaceuticals used in PET, such as Fluorine-18, have relatively short half-lives (around 110 minutes), ensuring that the patient is exposed to radiation for only a limited period. Second, the half-life influences logistical aspects, such as the timing of imaging sessions and the distribution of radiopharmaceuticals from production facilities (often cyclotrons) to hospitals or imaging centers. Understanding half-life also helps in planning for the safe disposal of radioactive materials after they are no longer useful for imaging or therapy.

The concept of half-life not only applies to radiopharmaceuticals but is also fundamental in radiation therapy, where precise calculations are needed to determine the duration of exposure to radioactive sources in treatments for cancer. By knowing the half-life of a radioactive isotope, physicians can calculate the optimal dosing schedule, balancing the need to deliver a sufficient therapeutic dose while minimizing harm to surrounding healthy tissues. This scientific measure allows for the meticulous planning and standardization of treatment protocols, making it a cornerstone of patient safety and efficacy in nuclear medicine.

Another example of standard scientific terminology in nuclear medicine is the **Becquerel (Bq)**, the SI unit of radioactivity, which measures the rate of radioactive decay in a given substance. Named after Henri Becquerel, who discovered radioactivity, one Becquerel corresponds to one decay per second. The adoption of this unit across the globe allows for uniformity in measuring and reporting levels of radioactivity, ensuring that all healthcare providers and researchers use a common language when assessing the activity of radiopharmaceuticals. This universality is vital in a field where precise dosimetry (the measurement of radiation doses) is required to ensure both the efficacy and safety of diagnostic procedures and treatments. In nuclear medicine, dosimetry calculations are based on the activity of the administered radiopharmaceutical, as measured in Becquerel’s, which directly affects patient management, regulatory compliance, and safety protocols. By using a standard unit like the Becquerel, professionals can communicate activity levels consistently, whether they are ordering radiopharmaceuticals from a supplier, conducting research, or treating patients.

Similarly, the **Gray (Gy)**, another SI unit, measures the absorbed dose of radiation, representing the amount of radiation energy absorbed by a specific mass of tissue. This unit is particularly important in radiation therapy, where precise dosages of radiation must be delivered to target cancerous tissues while minimizing exposure to surrounding healthy areas. The gray is used to quantify the energy deposited by ionizing radiation, allowing for standardized treatment plans across different medical institutions and ensuring that patient outcomes can be compared and studied with consistency. The universality of the gray helps to establish treatment thresholds, guide clinical decisions, and ensure that patients around the world receive comparable levels of care.

In addition to units like the Becquerel and gray, **Sieverts (Sv)** are used to express the biological effect of ionizing radiation on human tissue, known as the equivalent dose. The Sievert takes into account not only the absorbed dose but also the type of radiation and the biological sensitivity of the tissues exposed, providing a more comprehensive measure of the potential health risks associated with radiation exposure. This is crucial in nuclear medicine, where both patients and healthcare workers may be exposed to varying levels of radiation during imaging or therapy procedures. The Sievert enables practitioners to calculate and monitor radiation doses to ensure they remain within safe limits, thereby protecting patients and healthcare providers alike from the harmful effects of ionizing radiation. Once again, the use of an internationally recognized unit allows for consistent monitoring and reporting of radiation exposure, facilitating global standards for radiation safety.

Beyond specific units of measurement, nuclear medicine also employs standardized scientific terminology that draws on established principles from physics, chemistry, and biology. For example, terms such as **isotope**, **radiotracer**, and **attenuation** are used across different modalities and contexts, ensuring that professionals in the field can easily understand and apply the concepts regardless of their specific area of expertise. **Isotopes**, for instance, are atoms with the same number of protons but a different number of neutrons, and they are central to the production of radiopharmaceuticals used in PET and SPECT imaging. **Attenuation**, the reduction of radiation intensity as it passes through tissue, is a key concept in both imaging and radiation therapy, influencing how images are reconstructed and how radiation doses are calculated. These terms, based on well-established scientific principles, create a shared vocabulary that facilitates collaboration between physicists, radiologists, nuclear medicine technologists, and other healthcare professionals.

### Fusion of technologies

In the rapidly evolving landscape of nuclear medicine, the fusion of various imaging technologies has led to the creation of hybrid systems that enhance diagnostic capabilities and improve patient care. This amalgamation of different methodologies allows for a more comprehensive assessment of medical conditions by integrating distinct modalities that offer unique insights into physiological processes. As these technologies converge, new hybrid terms emerge to describe the integrated systems, providing healthcare professionals with a clearer understanding of the tools at their disposal. The development of hybrid imaging techniques not only reflects technological advancements but also underscores the importance of interdisciplinary collaboration in the pursuit of better diagnostic and therapeutic outcomes.

A prominent example of this fusion is the **PET/MRI** system, which combines **Positron Emission Tomography (PET)** with **Magnetic Resonance Imaging (MRI)** to provide both metabolic and anatomical information in a single scan. PET, as a nuclear medicine imaging technique, utilizes radiopharmaceuticals to visualize metabolic activity within the body, while MRI offers detailed structural images based on the magnetic properties of tissues. By merging these two modalities, PET/MRI enables clinicians to correlate functional data (metabolism) with anatomical data (structure) in a seamless manner, thereby enhancing diagnostic accuracy and treatment planning.

The clinical implications of PET/MRI are significant. For instance, in oncology, PET scans can identify areas of increased metabolic activity that may indicate malignancy, while MRI can delineate the precise anatomical boundaries of tumors. This combination is particularly beneficial for characterizing tumors, determining their stage, and planning treatment strategies. Moreover, the integration of these modalities can help differentiate between benign and malignant lesions, allowing for more targeted biopsies and interventions. By utilizing both metabolic and structural information, healthcare providers can develop more personalized treatment plans, ultimately improving patient outcomes.

Another important aspect of PET/MRI is its potential to reduce radiation exposure compared to traditional imaging methods that rely solely on PET/CT (Computed Tomography) systems. While both PET/CT and PET/MRI provide valuable information, the latter’s ability to visualize soft tissues with high resolution without ionizing radiation makes it particularly advantageous for certain patient populations, such as children or individuals requiring multiple imaging sessions. The ability to obtain comprehensive diagnostic information while minimizing radiation exposure aligns with the principles of patient safety and care, emphasizing the importance of thoughtful technology integration in modern medical practice.

The fusion of PET and MRI also exemplifies the interdisciplinary collaboration between physicists, radiologists, and nuclear medicine specialists. Each group brings its expertise to the development and refinement of hybrid imaging systems, ensuring that the resulting technologies are not only effective but also clinically relevant. This collaborative approach fosters innovation, leading to the creation of new techniques and applications that can further advance the field of nuclear medicine.

Beyond PET/MRI, the fusion of technologies extends to other hybrid systems, such as **SPECT/CT** (Single Photon Emission Computed Tomography/Computed Tomography). Like PET/MRI, SPECT/CT combines functional imaging from SPECT with high-resolution anatomical imaging from CT. This integration enhances the localization of abnormal metabolic activity, aiding in accurate diagnosis and treatment planning. SPECT/CT has proven particularly useful in various clinical settings, including cardiology, oncology, and neurology, as it allows for better correlation of functional data with anatomical structures, leading to more precise interventions.

In addition to improving diagnostic capabilities, the fusion of technologies often leads to the development of new algorithms and software that enhance image reconstruction and analysis. For example, hybrid imaging systems like PET/MRI and SPECT/CT require sophisticated computational techniques to accurately combine data from different modalities. Advances in image processing algorithms can improve the quality of reconstructed images, enhance contrast resolution, and reduce artifacts, resulting in more reliable diagnostic information. This technological evolution demonstrates how the fusion of methodologies not only enriches imaging capabilities but also drives research and development in the field of nuclear medicine.

The emergence of hybrid terms, such as PET/MRI and SPECT/CT, also reflects a shift in the language of nuclear medicine, emphasizing the importance of integrated approaches to patient care. As these technologies become more commonplace, understanding their applications and implications becomes essential for healthcare professionals across disciplines. By using hybrid terminology, practitioners can communicate the advantages and capabilities of these integrated systems effectively, fostering collaboration among specialists and improving overall patient management.

Furthermore, the fusion of technologies can pave the way for future innovations in nuclear medicine. As research continues to explore new combinations of imaging modalities, such as **ultrasound** or **optical imaging**, we may see the development of even more sophisticated hybrid systems that enhance diagnostic precision and broaden the scope of clinical applications. These advancements highlight the dynamic nature of the field, where the integration of diverse technologies can lead to novel approaches in diagnosis, treatment, and monitoring of diseases.

### Numerical and statistical descriptors

In the realm of nuclear medicine, the use of numerical and statistical descriptors plays a vital role in interpreting and standardizing imaging results, ultimately facilitating more effective patient management and treatment decisions. These terms, grounded in statistical analysis, represent calculated values derived from the imaging process and provide a means to quantify biological activity, thus allowing for a more objective assessment of metabolic processes. By employing standardized metrics, healthcare professionals can uniformly interpret imaging findings across diverse patient populations and clinical contexts, improving the consistency and reliability of diagnostic evaluations.

One of the most prominent examples of such a statistical descriptor is the **Standardized Uptake Value (SUV)**. The SUV quantifies the uptake of radiopharmaceuticals in specific tissues relative to the average uptake in the whole body, often normalized to body weight or lean body mass. This ratio provides a measure of metabolic activity within a particular region, enabling clinicians to assess the presence and aggressiveness of various conditions, such as tumors. By standardizing the uptake measurement, the SUV facilitates comparisons between different patients and imaging sessions, allowing for a more robust evaluation of treatment response or disease progression.

The calculation of SUV involves the use of positron emission tomography (PET) data, where the concentration of the radiotracer in a region of interest (ROI) is measured and divided by the injected dose and body weight or lean body mass. This mathematical approach allows for the elimination of variability due to differences in radiotracer distribution, patient size, and metabolic rates, leading to a more standardized interpretation of PET results. The ability to compare SUVs across patients and studies enhances the overall reliability of the findings, supporting more accurate clinical decision-making.

The importance of SUV extends beyond oncology; it is also valuable in other clinical scenarios, including cardiac imaging and neurological assessments. For example, in cardiac PET imaging, SUV can be used to evaluate myocardial perfusion and viability, providing insights into ischemic heart disease. Similarly, in neuroimaging, changes in SUV can help assess neurodegenerative disorders, allowing clinicians to track the progression of diseases like Alzheimer’s or Parkinson’s. The applicability of SUV across various medical disciplines underscores its significance as a standard metric in nuclear medicine.

Moreover, the use of numerical and statistical descriptors such as SUV is essential for clinical trials and research studies. These standardized measures provide a common language for reporting outcomes and comparing results across different studies, which is critical for meta-analyses and systematic reviews. By adopting a uniform approach to quantifying radiotracer uptake, researchers can better evaluate the efficacy of new therapies and imaging techniques, leading to advancements in patient care and treatment protocols.

Another key statistical term in nuclear medicine is the **Mean Residence Time (MRT)**, which quantifies the average time that a radiopharmaceutical remains in a specific organ or tissue. MRT is particularly relevant in the context of radionuclide therapy, where understanding the distribution and retention of radioactive isotopes in target tissues is crucial for optimizing treatment efficacy and minimizing side effects. By integrating MRT calculations into treatment planning, clinicians can better predict the therapeutic outcomes and adjust dosing accordingly, ensuring a more personalized approach to patient care.

Furthermore, the application of statistical analysis extends to the evaluation of imaging protocols and the optimization of imaging techniques. By analyzing large datasets derived from clinical imaging, researchers can identify patterns, correlations, and potential confounding factors that may influence the interpretation of imaging results. This information is essential for refining imaging protocols, improving the accuracy of diagnostic criteria, and ensuring that radiopharmaceuticals are used effectively and safely.

In addition to SUV and MRT, other numerical descriptors such as **Volume of Distribution (Vd)** and **Time-Activity Curves (TAC)** provide further insights into the pharmacokinetics of radiopharmaceuticals. Vd reflects the extent to which a drug disperses throughout the body, offering valuable information on its distribution and potential efficacy. TAC, on the other hand, describes the relationship between radioactivity in a region over time, providing a dynamic view of how a radiotracer behaves within the body. These metrics, when combined with SUV, create a comprehensive picture of the radiopharmaceutical’s behavior, supporting more informed clinical decisions.

The reliance on numerical and statistical descriptors in nuclear medicine reflects a broader trend in medicine towards evidence-based practice, where data-driven approaches are employed to enhance patient outcomes. As imaging technology continues to advance and generate larger volumes of data, the need for standardized metrics and statistical analysis will only increase. By incorporating these descriptors into routine practice, nuclear medicine professionals can ensure that they are interpreting imaging results accurately and consistently, ultimately leading to improved patient care.

## Discussion

The systematic development of nuclear medicine terminology is essential for ensuring effective interdisciplinary collaboration, accessibility, and adaptability to technological advancements. By using classical linguistic roots, the language provides a universal framework that allows professionals across diverse disciplines to communicate clearly and efficiently. This structured approach ensures that complex concepts can be understood without ambiguity, fostering collaboration and enhancing the accuracy of both clinical and research-related communication.

The adaptability of terminology further supports the integration of emerging technologies, particularly hybrid imaging systems that combine functional and anatomical capabilities. These advancements necessitate the creation of terms that effectively reflect the evolution of the field while maintaining clarity and precision. Standardized units and descriptors promote consistency, enabling seamless collaboration across international and multidisciplinary teams, and ensuring uniformity in research and clinical practices.

The historical aspect of terminology adds another dimension, connecting modern innovations to their foundational roots and acknowledging the contributions of key individuals. This connection to the past enriches the language of the field, inspiring future progress while preserving the legacy of its pioneers. Collectively, these linguistic strategies create a dynamic and resilient framework that not only meets the current needs of nuclear medicine but also paves the way for future advancements.

In conclusion, the structured development of nuclear medicine terminology is vital for advancing the field. Through a combination of linguistic precision, global standardization, and adaptability, the language ensures effective communication and collaboration across disciplines. This robust framework supports the integration of innovative technologies, fosters international cooperation, and enhances the clarity needed for high-quality patient care and groundbreaking research. As nuclear medicine continues to evolve, its terminology will remain a cornerstone of progress, enabling the field to meet future challenges and opportunities effectively.

## Results

The methodology for forming terms in nuclear medicine employs a comprehensive and systematic approach that incorporates various linguistic and scientific principles. The use of Greek and Latin roots provides a universal framework, enabling precise communication of complex concepts related to imaging techniques and subatomic particles. Scientific prefixes and suffixes contribute to the creation of clear and descriptive terminology, ensuring consistency across fields and facilitating interdisciplinary collaboration. Abbreviations and acronyms, such as PET and SUV, enhance communication efficiency by condensing complex terminology into recognizable forms, crucial for a fast-paced and technical discipline. Descriptive compounding creates intuitive terms that effectively communicate the purpose and mechanisms of technologies, fostering understanding among professionals. Additionally, naming technologies after their discoverers honors individual contributions and underscores the historical context of scientific advancements. The adoption of standard scientific units and terminology ensures accuracy and consistency in measurements, while the fusion of technologies, as seen in hybrid systems like PET/MRI, enhances diagnostic capabilities by combining functional and anatomical imaging. Numerical and statistical descriptors, such as Standardized Uptake Value (SUV), provide a standardized framework for interpreting results and improving clinical decision-making. Collectively, these approaches ensure that the terminology in nuclear medicine remains clear, accurate, and universally applicable, ultimately advancing communication, collaboration, and patient care in the field as it evolves.

## Ethics and consent

No ethics and consent were required.

## Data Availability

No data are associated with this article.
