# Supplementary material for: Methods of terms formation in nuclear medicine
Source: Open Res Eur. 2025 Feb 10;5:46. [Version 1] doi: 10.12688/openreseurope.18941.1 (PMC11894368; doi:10.12688/openreseurope.18941.1)
Supplement: Peer review history

# Reviewer report: Reviewer response for version 1

**DOI:** 10.21956/openreseurope.20493.r51352

**Published:** 2025-03-10

**Authors:** Dorina Macovei

**Affiliations:** 1 Moldova State University, Chisinau, Moldova

This article discusses the methods of forming terminology in nuclear medicine, emphasizing the importance of clear communication in this complex field. It highlights the use of Greek and Latin roots, scientific prefixes and suffixes, abbreviations, and descriptive compounding to create precise and universally understandable terms. The article concludes that this systematic terminology enhances global collaboration and innovation, ultimately advancing patient care.

 This work demonstrates a clear and accurate presentation. The study design is appropriate, displaying academic merit and providing sufficient methodological and analytical detail to enable replication. Furthermore, source data underlying the results is available, ensuring full reproducibility. In conclusion, the results adequately support the conclusions drawn, confirming the robust nature of this scholarly endeavor.

 At the same time, the writers should revise the following aspects:

 1) too much specialized description/explanation of terms is provided, which is irrelevant for linguistic research;

 2) 'pharmaceutical' is not a suffix;

 3) introduce more citations.

 I would recommend accepting the article after addressing these points.

Is the study design appropriate and does the work have academic merit?

Yes

Is the work clearly and accurately presented and does it cite the current literature?

Yes

If applicable, is the statistical analysis and its interpretation appropriate?

Partly

Are all the source data underlying the results available to ensure full reproducibility?

Yes

Are the conclusions drawn adequately supported by the results?

Yes

Are sufficient details of methods and analysis provided to allow replication by others?

Yes

Reviewer Expertise:

Terminology

I confirm that I have read this submission and believe that I have an appropriate level of expertise to confirm that it is of an acceptable scientific standard.

# Reviewer report: Reviewer response for version 1

**DOI:** 10.21956/openreseurope.20493.r51142

**Published:** 2025-02-24

**Authors:** Azer Sadigov, Jalal Naghiyev

**Affiliations:** 1 Department of Nuclear Research, Innovation and Digital Development Agency, Baku, Baku, Azerbaijan; 2 Institute of Radiation Problems- Ministry of Science and Education, Baku, Azerbaijan; 3 Nuclear Research Department, Innovation and Digital Development Agency, Baku, Baku, Azerbaijan

Nuclear medicine is a rapidly developing interdisciplinary field that utilizes radioactive substances for diagnostics, therapy, and scientific research. The continuous advancement of this domain is accompanied by the emergence of new technologies, imaging methods, and radiopharmaceuticals, necessitating precise and standardized terminology.

 - Is the work clearly and accurately presented and does it cite the current literature? 

 The article does cite foundational and historical references; however, more recent and cutting-edge publications on nuclear medicine innovations should be incorporated. The following areas require additional references:

 The role of deep learning in automated radiotracer quantification.

 Recent developments in hybrid imaging systems and their clinical impact.

 The inclusion of more up-to-date references would strengthen the article's scientific foundation.

 -Are sufficient details of methods and analysis provided to allow replication by others?

 While the article provides a general discussion on terminology formation, it lacks detailed methodology and analytical frameworks that would allow full replication of its approach. Suggested improvements include:

 Providing a step-by-step framework for analyzing medical terminology.

 Offering examples of terminology evolution based on technological advancements.

 Citing specific linguistic models used in medical terminology research.

Is the study design appropriate and does the work have academic merit?

Yes

Is the work clearly and accurately presented and does it cite the current literature?

Partly

If applicable, is the statistical analysis and its interpretation appropriate?

Not applicable

Are all the source data underlying the results available to ensure full reproducibility?

Yes

Are the conclusions drawn adequately supported by the results?

Yes

Are sufficient details of methods and analysis provided to allow replication by others?

Partly

Reviewer Expertise:

My areas of research: Radiation applications, nuclear physics, nuclear medicine and engineering

We confirm that we have read this submission and believe that we have an appropriate level of expertise to confirm that it is of an acceptable scientific standard.
